# Supplementary material for: Greater Consumption of Total and Individual Lignans and Dietary Fibers Were Significantly Associated with Lowered Risk of Hip Fracture—A 1:1 Matched Case–Control Study among Chinese Elderly Men and Women
Source: Nutrients. 2022 Mar 5;14(5):1100. doi: 10.3390/nu14051100 (PMC8912333; doi:10.3390/nu14051100)
Supplement: Supplementary file 1 [file nutrients-14-01100-s001.zip › nutrients-1617526-supplementary.pdf]

Table S1: The main food sources of dietary intake of lignans and its four subclasses in Chinese elderly men and women, Guangzhou, China

	Mean±SD	Median(quartile range)	Main food sources (%)
Total lignans (μg/d)	469.8±898.5	375.8 (255.3, 532.8)	Vegetables (56.7%), nuts (22.8%), cereals (9.6%), legumes (4.4%), fruits (3.6%), tea (2.3%) and milk (0.5%).
Individual lignans(μg/d)			
MAT(μg/d)	4.6±12.7	3.3 (2.0, 5.3)	Nuts (37.5%), vegetables (20.8%), fruits (15.6%), cereals (12.2%), legumes (6.4%), milk (5.2%) and tea (2.2%)
LARI(μg/d)	183.5±164.6	160.0 (117.2, 216.1)	Vegetables (73.8%), cereals (11.8%), nuts (8.2%), legumes (3.6%), fruits (2.1%), milk (0.3%) and tea (0%)
PINO(μg/d)	226.7±682.4	152.6 (90.8, 264.0)	Vegetables (49.1%), nuts (40.4%), fruits (4.1%), legumes (2.1%), cereals (3.5%), tea (0.6%) and milk (0.2%)
SECO(μg/d)	55.8±88.1	43.8(29.2, 61.8)	Vegetables (30.5%), cereals (23.0%), tea (14.9%), legumes (14.0%), nuts (9.3%), fruits (6.1%) and milk (1.4%)

Dietary total and individual lignans were presented as both mean ± standard deviation and median (quartile range). The percentages of food sources for total and individual lignans were calculated as the amount (μg/d) of lignans intake from individual food sources divided by lignans amounts from all food sources, and then multiply 100. MAT: matairesinol; SECO: secoisolariciresinol; PINO: pinoresinol; LARI: lariciresinol.
